# Presence of autoantibodies in “seronegative” rheumatoid arthritis associates with classical risk factors and high disease activity

**DOI:** 10.1186/s13075-020-02191-2

**Published:** 2020-07-16

**Authors:** Evan Reed, Anna Karin Hedström, Monika Hansson, Linda Mathsson-Alm, Boel Brynedal, Saedis Saevarsdottir, Martin Cornillet, Per-Johan Jakobsson, Rikard Holmdahl, Karl Skriner, Guy Serre, Lars Alfredsson, Johan Rönnelid, Karin Lundberg

**Affiliations:** 1 Division of Rheumatology, Department of Medicine Solna, Karolinska Institutet, Karolinska University Hospital, CMM L8:04, 171 76 Stockholm, Sweden; 2 Institute of Environmental Medicine, Karolinska Institutet, Stockholm, Sweden; 3 Department of Clinical Neuroscience, Karolinska Institutet, Stockholm, Sweden; 4 Thermo Fisher Scientific, Uppsala, Sweden; 5 Department of Immunology Genetics and Pathology, Uppsala University, Uppsala, Sweden; 6 Division of Clinical Epidemiology, Department of Medicine Solna, Karolinska Institutet, Stockholm, Sweden; 7 Faculty of Medicine, School of Health Sciences, University of Iceland, Reykjavik, Iceland; 8 Unité Différenciation Epithéliale et Autoimmunité Rhumatoïde, Université de Toulouse-INSERM UMR 1056, Toulouse, France; 9 Section for Medical Inflammation Research, Department of Medical Biochemistry and Biophysics, Karolinska Institutet, Stockholm, Sweden; 10 Department of Rheumatology and Clinical Immunology, Charité University, Berlin, Germany; 11 Centre for Occupational and Environmental Medicine, Region Stockholm, Stockholm, Sweden

**Keywords:** Rheumatoid arthritis (RA), Autoantibodies, Anti-citrullinated protein antibodies (ACPA), Cyclic citrullinated peptide (CCP), Rheumatoid factor (RF), Anti-carbamylated protein antibodies (anti-CarP), Disease activity score for 28 joints (DAS28), HLA-DRB1 shared epitope (SE), Smoking

## Abstract

**Background:**

Rheumatoid arthritis (RA) is classified as seropositive or seronegative, depending on the presence/absence of rheumatoid factor (RF), primarily IgM RF, and/or anti-citrullinated protein antibodies (ACPA), commonly detected using anti-cyclic citrullinated peptide (CCP) assays. Known risk factors associate with the more severe seropositive form of RA; less is known about seronegative RA. Here, we examine risk factors and clinical phenotypes in relation to presence of autoantibodies in the RA subset that is traditionally defined as seronegative.

**Methods:**

Anti-CCP2 IgG, 19 ACPA fine-specificities, IgM/IgG/IgA RF, anti-carbamylated-protein (CarP) antibodies, and 17 other autoantibodies, were analysed in 2755 RA patients and 370 controls. Antibody prevalence, levels, and co-occurrence were examined, and associations with risk factors and disease activity during 5 years were investigated for different antibody-defined RA subsets.

**Results:**

Autoantibodies were detected in a substantial proportion of the traditionally defined seronegative RA subset, with ACPA fine-specificities found in 30%, IgA/IgG RF in 9.4%, and anti-CarP antibodies in 16%, with a 9.6% co-occurrence of at least two types of RA-associated autoantibodies. HLA-DRB1 shared epitope (SE) associated with the presence of ACPA in anti-CCP2-negative RA; in anti-CCP2-positive RA, the SE association was defined by six ACPA fine-specificities with high co-occurrence. Smoking associated with RF, but not with ACPA, in anti-CCP2-negative RA. Presence of ACPA and RF, but not anti-CarP antibodies, in conventionally defined “seronegative” RA, associated with worse clinical outcome.

**Conclusions:**

“Seronegative” RA is not truly a seronegative disease subset. Additional screening for ACPA fine-specificities and IgA/IgG RF defines a group of patients that resembles seropositive patients with respect to risk factors and clinical picture and may contribute to earlier diagnosis for a subset of anti-CCP2−/IgM RF− patients with a high need for active treatment.

## Introduction

Presence of anti-citrullinated protein antibodies (ACPA) is a hallmark of rheumatoid arthritis (RA), and together with rheumatoid factor (RF), part of the 2010 American College of Rheumatology (ACR)/European League Against Rheumatism (EULAR) classification criteria [1]. Patients positive for ACPA and/or RF may be referred to as “seropositive” and make up approximately two thirds of the RA population. Seropositive and seronegative RA seem to have disparate mechanisms in predisposition, since HLA-DRB1 shared epitope (SE) alleles, as well as a number of other genes, associate primarily with ACPA-positive RA [2, 3], while other genes have been linked to ACPA-negative disease [4], suggesting that these subsets are partly separate disease entities.

While typically considered having a less inflammatory and less destructive form of RA [5], seronegative patients require more clinical symptoms to be classified as having RA according to the 2010 ACR/EULAR criteria [1], compared to seropositive patients, and may consequently be diagnosed later [6]. Since all patients benefit from early treatment, early diagnosis is critical also for seronegative patients [7–9]. A major obstacle to this is the lack of biomarkers.

ACPA status is commonly determined using assays based on cyclic citrullinated peptides (CCP), such as the “second generation” CCP2 test. However, these tests utilise synthetic peptides as surrogate markers for in vivo citrullinated antigens, while recent years have seen a number of studies describing ACPA targets on defined proteins (e.g. citrullinated fibrinogen [10], vimentin [11], collagen type II (CII) [12], α-enolase [13], histones [14], and tenascin C [15]). Since the CCP2 test does not capture all ACPA, multiplex assays capable of detecting multiple ACPA fine-specificities simultaneously may be useful, as more patients could potentially be categorised as ACPA-positive/seropositive [14, 16–19]. Using such a multiplex citrullinated peptide array, we have recently shown that 16% of anti-CCP2-negative RA patients were in fact ACPA-positive [19].

The other major autoantibody in RA, RF, is most often defined as IgM directed against the constant region of IgG, although other RF isotypes (mainly IgG and IgA) are also present in subsets of patients, but generally not screened for in the clinic [20]. More recently, antibodies to carbamylated proteins (anti-CarP antibodies) have been described in RA [21], and additional autoantibodies, including anti-nuclear antibodies, which are primarily linked to other rheumatic/autoimmune conditions, have also been detected in subsets of RA [22].

In this study, we have used the Swedish population-based RA case-control study EIRA (Epidemiological Investigation of RA) as a basis for the analysis of autoantibodies present in the RA subset conventionally defined as seronegative, and we have investigated whether the presence of such antibodies associates with classical RA risk factors and have predictive value for disease activity.

## Patients and methods

### Study population

This study includes newly diagnosed RA based on the 1987 revised ACR criteria [23] and age-, sex-, and residential area-matched controls from the Swedish population-based EIRA cohort [24]. Information on smoking was collected via self-reported questionnaire at baseline; individuals were categorised as “ever smokers” (including current and former smokers) or “never smokers”. Patients and controls donated blood at inclusion and were genotyped for HLA-DRB1 alleles and PTPN22 (rs2476601) polymorphism as previously described [25, 26]. HLA-DRB1*01 (except DRB1*0103), *04, and *10 were classified as SE. Data on smoking and genetics were available for 2198 RA patients and 2797 controls. Baseline disease activity score for 28 joints (DAS28) and C-reactive protein (CRP) levels (mg/L) were captured on 1986 and 2096 RA patients, respectively, by linking EIRA with the Swedish Rheumatology Quality register [27]. Five-year clinical follow-up data (DAS28-CRP) was analysed in 1086 RA patients.

This study has been performed in compliance with the Declaration of Helsinki, with informed consent obtained from all participants, and ethical approval granted at the Regional Ethical Review Board in Stockholm.

### Antibody detection: multiplex microarray and ELISA

High-throughput IgG screening was performed on sera from 2755 RA cases and 370 controls using a custom-made microarray chip (Thermo Fisher Scientific, ImmunoDiagnostics), as previously described [18]. Antigens included 19 citrullinated peptides (and arginine-containing equivalents) from filaggrin, fibrinogen, vimentin, α-enolase, collagen type II (CII), and heterogeneous nuclear ribonucleoprotein-A3 (hnRNP-A3), and 17 non-citrullinated antigens reported as autoantibody targets in other rheumatic/autoimmune disorders (Additional files 1 and 2). Cutoff (for each antibody) was set based on reactivity among controls and correspond to the highest 98th percentile found in 20 randomly selected subsets comprising 80% of the control population.

Anti-CCP2 IgG was measured using Immunoscan CCPlus® ELISA (Euro-Diagnostica AB, Malmö, Sweden), according to the manufacturer’s instructions, with a cutoff of 25 U/mL. IgM, IgG, and IgA RF were analysed using EliA™ immunoassay on Phadia 2500 (Phadia AB, Uppsala, Sweden), using cutoff values as stated in the manufacturer’s instructions. Anti-carbamylated fibrinogen antibodies were measured in a subset of patients (*n* = 1944), as previously reported [28], with cutoff set at the 98th percentile among 316 EIRA controls.

### Statistical methods

Differences in antibody levels/prevalence and DAS28-CRP were analysed by Mann-Whitney *U* test. Co-occurrence of ACPA and comparison of correlation coefficients between different ACPA fine-specificities in anti-CCP2-positive and anti-CCP2-negative RA were calculated using Pearson’s correlation (Rv.3.3.3), among patients that were positive for at least one ACPA fine-specificity. Associations between RA subsets and risk factors were determined by unconditional logistic regression and presented as odds ratios (OR) with 95% confidence intervals (CI) (SAS 9.4). Analyses were adjusted for age, sex, and residential area, and PTPN22, smoking, and SE when appropriate.

## Results

### Comparison of ACPA fine-specificities in anti-CCP2-positive and anti-CCP2-negative RA

EIRA cases were first divided based on anti-CCP2 IgG status, and anti-CCP2-positive patients were younger, more frequently smokers, and carriers of HLA-DRB1 SE and PTPN22 rs2476601, while there were no differences with regard to baseline DAS28, CRP, or the female-to-male ratio, as compared to anti-CCP2-negative patients (Additional file 3).

**Fig. 1:**
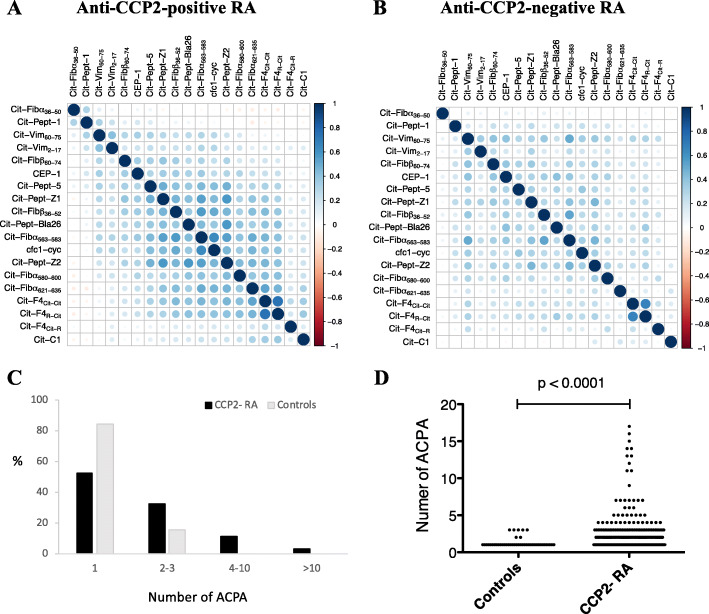
Co-occurrence of ACPA fine-specificities. **a**, **b** Correlation plots illustrating co-occurrence of different ACPA fine-specificities, in anti-CCP2-positive and anti-CCP2-negative RA. Correlation (Pearson *r*^2^) between binary antibody expression vectors, among patients that were positive for at least one ACPA fine-specificity, are shown; plotted using corrplot (v.0.77). The degree of correlation is illustrated in colours, according to Pearson’s correlation coefficient (scale shown on the right). **c**, **d** The number of ACPA fine-specificities in anti-CCP2-negative RA and controls. The frequency (%) of anti-CCP2-negative RA patients and controls with 1, 2–3, 4–10, or > 10 ACPA fine-specificities, and the number of ACPA per individual patient/control, are shown. Only ACPA-positive individuals with higher reactivity against citrulline compared to arginine-containing peptides were included in the analysis

As we have recently shown, using the multiplex citrullinated peptide array, ACPA fine-specificities can be detected in a substantial proportion (16%) of the anti-CCP2-negative EIRA RA population [19], also in line with previous data [16, 17]. In this extended analysis, we show that the pattern of citrulline-reactivity is similar for anti-CCP2-negative and anti-CCP2-positive RA, albeit with lower prevalence, levels, and co-occurrence of ACPA fine-specificities. Eleven out of 19 ACPA fine-specificities were detected in anti-CCP2-negative RA, in frequencies significantly above controls, while all 19 ACPA were detected in anti-CCP2-positive RA (Table 1). The citrullinated fibrinogen-derived peptide Cit-Fibß_60–74_ was the most commonly detected fine-specificity in both subsets, followed by Cit-peptide-5 and Cit-peptide-Z1 derived from citrullinated hnRNP-A3 and Cit-Fibß_36–52_ from fibrinogen. ACPA levels among ACPA fine-specificity positive individuals were higher in anti-CCP2-positive RA, compared to anti-CCP2-negative RA (Table 1), and in anti-CCP2-negative RA, compared to controls (Additional file 4). Co-occurrence of different ACPA fine-specificities showed a similar correlation profile for anti-CCP2-positive and anti-CCP2-negative subsets (*r*^2^ = 0.65, *p* = 5.4e−22), with high correlation between most ACPA, but an independent expression pattern for some (e.g. Cit-Fibα_36–50_, Cit-Peptide-1, Cit-F4_Cit-R_, and Cit-C1) (Fig. 1a, b). Co-occurrence of multiple ACPA was rare among controls; the majority (84.1%) had only one ACPA fine-specificity, and none of the controls had more than three. The number of citrullinated peptides recognised by control sera was significantly lower than that of anti-CCP2-negative RA sera, where 15% were positive for more than three ACPA, and 32.7% were positive for two or three fine-specificities (Fig. 1c, d).

**Table 1:** ACPA fine-specificities in anti-CCP2+ and anti-CCP- RA and controls

ACPA fine-specificities	Antibody frequencies (%)				Antibody levels (median)^a^		
	CCP2+	CCP2−	Controls	*p* value^b^	CCP2+	CCP2−	*p* value^c^
Cit-Fibß_60–74_	80.6	9.5	0.8	**< 0.0001**	195.3	27.1	**< 0.0001**
Cit-peptide-5	75.4	8.6	1.1	**< 0.0001**	81.4	27.2	**< 0.0001**
Cit-peptide-Z1	69.3	5.1	0.5	**0.0001**	167.4	38.6	**< 0.0001**
Cit-Fibß_36–52_	65.6	5.0	1.4	**0.003**	605.0	186.4	**< 0.0001**
Cit-Vim_60–75_	63.5	2.9	1.1	**0.049**	641.6	321.9	**0.009**
CEP-1	60.9	4.4	1.1	**0.003**	678.2	316.1	**< 0.0001**
cfc1-cyc (CCP1)	59.9	3.8	1.1	**0.009**	514.7	166.0	**< 0.0001**
Cit-Fibα_563–583_	57.6	2.5	1.1	0.10	444.8	144.0	**0.002**
Cit-peptide-Z2	49.5	2.9	0.3	**0.003**	150.9	58.6	**0.002**
Cit-peptide-1	45.0	4.7	1.9	**0.02**	72.7	29.2	**< 0.0001**
Cit-Fibα_621–635_	42.9	4.6	1.4	**0.005**	384.0	123.0	**< 0.0001**
Cit-peptide-Bla26	39.9	4.4	1.4	**0.006**	80.3	48.7	**0.003**
Cit-Vim_2–17_	40.3	2.6	1.4	0.16	75.9	72.8	0.712
Cit-F4_(Cit-Cit)_	36.1	3.4	1.6	0.08	84.9	60.6	**0.032**
Cit-F4_(R-Cit)_	29.9	2.1	1.4	0.36	86.4	80.6	0.366
Cit-Fibα_580–600_	24.2	2.8	1.4	0.11	264.2	197.2	**0.024**
Cit-Fibα_36–50_	17.6	4.0	1.9	0.053	422.9	265.5	**< 0.0001**
Cit-C1	10.8	1.9	1.4	0.48	69.3	87.9	0.383
Cit-F4_(Cit-R)_	6.2	1.8	1.1	0.34	403.0	306.5	0.382

^a^Median antibody levels are shown for ACPA+ patients only. *P* values indicate differences between ^b^anti-CCP2− RA and controls or between ^c^anti-CCP2+ and anti-CCP2− RA

### Presence of RF in anti-CCP2-positive and anti-CCP2-negative RA

**Fig. 2:**
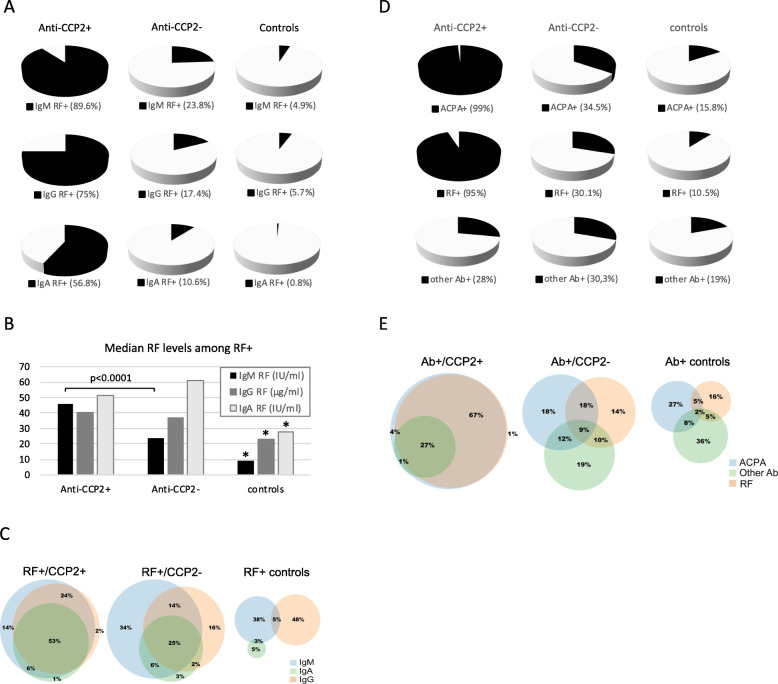
Autoantibody data in anti-CCP2-positive RA, anti-CCP2-negative RA, and controls. **a** Prevalence of IgM, IgG, and IgA RF. **b** Median IgM, IgG, and IgA RF levels among RF-positive individuals; asterisks (*) indicate significantly lower IgM, IgG, and IgA RF levels in controls compared to RA patients (*p* < 0.0001 for all). **c** Co-occurrence of different RF isotypes among RF-positive individuals, in anti-CCP2-positive RA (*n* = 1655/1766), anti-CCP2-negative RA (*n* = 298/989), and controls (*n* = 39/370). **d** Prevalence of different autoantibody groups (ACPA, RF, and other autoantibodies). **e **Co-occurrence of different autoantibody groups among autoantibody-positive individuals, in anti-CCP2-positive RA (*n* = 1753/1766), anti-CCP2-negative RA (*n* = 591/989), and controls (*n* = 137/370)

In the present study, we have also analysed RF isotypes IgM, IgG, and IgA in EIRA, and all RF isotypes were detected in the anti-CCP2-negative subset, with IgM RF present in 23.8%, IgG RF in 17.4%, and IgA RF in 10.6% (Fig. 2a). Higher frequencies were detected in anti-CCP2-positive RA: 89.6% (IgM RF), 75% (IgG RF), and 56.8% (IgA RF). IgM RF levels were higher in anti-CCP2-positive, compared to anti-CCP2-negative patients, while no differences were observed for IgG and IgA RF levels (Fig. 2b). Co-occurrence of at least two RF isotypes was 47% among RF+/anti-CCP2− patients, and all three isotypes were present in 25%; the corresponding figures for RF+/anti-CCP2+ patients were 83% and 53%, respectively (Fig. 2c). Co-occurrence of RF isotypes was rare among RF-positive controls, and RF levels were significantly lower than in patients.

### Presence of other autoantibodies in anti-CCP2-positive and anti-CCP2-negative RA

In addition to ACPA fine-specificities and RF isotypes, we have screened for the presence of 17 autoantibodies primarily associated with other autoimmune rheumatic diseases. In general, these other autoantibodies were present at low frequencies in EIRA, with small differences between anti-CCP2-positive and anti-CCP2-negative patients (Additional file 5). Six out of 17 (Ro60/SSA, Ro52/SSA, PMScl100, La/SSB, U1-RNP-C, and CENPB) were detected at significantly higher frequencies in RA compared to controls, with antibodies against Ro60/SSA and Ro52/SSA as the most common, both with a frequency of 5.3% in anti-CCP2-negative RA and 4.8% and 3.4%, respectively, in anti-CCP2-positive RA.

### Narrowing the “serological gap”

When combining ACPA, RF, and other autoantibodies, we detect autoantibodies in 59.8% of the anti-CCP2-negative RA population, with ACPA fine-specificities found in 34.5%, RF isotypes in 30.1%, and other autoantibodies in 30.3%. Notably, considering the high number of autoantibodies investigated (*n* = 39), each with an individual cutoff set between the 95th and 98th percentile, autoantibodies were also detected in 37% of the control population, with ACPA in 15.8%, RF in 10.5%, and other autoantibodies in 19%. Importantly, co-occurrence of ACPA and RF was 15.7% in anti-CCP2-negative RA, compared to only 2.7% in controls. For an illustration of the distribution of ACPA, RF, and other autoantibodies in anti-CCP2-positive RA, anti-CCP2-negative RA, and controls, see Fig. 2d and e.

### Autoantibodies in relation to RA risk factors

Classical RA risk factors HLA-DRB1 SE, PTPN22 polymorphism, and cigarette smoking are known to associate with the anti-CCP2-positive subset of RA [26, 29]. In addition, we and others have shown associations between SE and the presence of ACPA in anti-CCP2-negative RA. Here we can confirm an association between SE and ACPA+/anti-CCP2− RA, while there was no significant association between SE and the presence of RF or other autoantibodies in anti-CCP2-negative RA (Table 2). Smoking, on the other hand, did not associate with ACPA, or other autoantibodies, but showed a significant association with RF in anti-CCP2-negative RA. PTPN22 polymorphism associated significantly with all anti-CCP2-negative RA subsets, irrespective of autoantibody status (Additional file 6).

**Table 2:** Associations between autoantibodies and risk factors, in anti-CCP2− RA

Subgroup	Exposure			
	**SE−**	**SE+**	**OR (95% CI)**^a^	**OR (95% CI)**^b^
Controls	1341	1456	1.0 (ref)	1.0 (ref)
ACPA−	246	265	0.99 (0.82–1.2)	1.00 (0.83–1.21)
ACPA+	126	176	**1.29** (1.01–1.64)	**1.30** (1.02–1.65)
RF−	258	293	1.04 (0.87–1.26)	1.05 (0.88–1.27)
RF+	114	148	1.20 (0.93–1.55)	1.21 (0.94–1.57)
Other Ab−	242	316	**1.20** (1.00–1.45)	**1.22** (1.01–1.46)
Other Ab+	130	125	0.88 (0.68–1.14)	0.89 (0.69–1.15)
	**Never smoker**	**Ever smoker**	**OR (95% CI)**^a^	**OR (95% CI)**^c^
Controls	1208	1589	1.0 (ref)	1.0 (ref)
ACPA−	184	327	**1.35** (1.11–1.64)	**1.34** (1.10–1.63)
ACPA+	121	181	1.16 (0.91–1.48)	1.15 (0.90–1.47)
RF−	221	330	1.13 (0.94–1.36)	1.13 (0.93–1.36)
RF+	84	178	**1.65** (1.26–2.16)	**1.63** (1.24–2.14)
other Ab-	203	355	**1.33** (1.10–1.60)	**1.32** (1.09–1.59)
other Ab+	102	153	1.16 (0.89–1.51)	1.16 (0.89–1.51)

Odds ratios were adjusted for ^a^age, sex, and residential area, ^b^PTPN22 and smoking, or ^c^SE and PTPN22. *ACPA* = any ACPA fine-specificity; *RF* = IgM and/or IgG and/or IgA RF; *other Ab* = any other autoantibody

Based on these data, we proceeded to investigate associations between SE and individual ACPA fine-specificities further, as well as associations between smoking and different RF isotypes, in both anti-CCP2-positive and anti-CCP2-negative RA subsets.

In anti-CCP2-positive RA, SE associated with all individual ACPA fine-specificities, in line with what we have previously shown in RA [19]. In our extended analysis, we found that the SE association was significantly stronger in the presence of ACPA reactive with Cit-Fibβ_60–74_, Cit-peptide-5, Cit-peptide-Z1, Cit-Vim_60–75_, CEP-1, and Cit-Vim_2–17_, than in the absence of these six ACPA fine-specificities (Additional file 7); two of these ACPA fine-specificities (Cit-Fibβ_60–74_ and Cit-Vim_60–75_) also showed significant associations with SE in anti-CCP2-negative RA.

The association with smoking was significantly stronger in the presence of IgG and IgA RF in anti-CCP2-positive RA and in the presence of IgM and IgA RF in anti-CCP2-negative RA, than in the absence of these RF isotypes (Additional file 8). Moreover, in a sub-analysis, comparing never, current, and former smokers in the whole RA population, we observed significantly higher RF levels (all isotypes) and anti-CCP2 IgG levels in current and former smokers, compared to never smokers (*p* < 0.0001) (Additional file 9). However, when comparing current to former smokers, only RF levels (all isotypes) were significantly elevated (*p* < 0.0001 for IgM and IgA RF; *p* = 0.0003 for IgG RF), while anti-CCP2 IgG levels (*p* = 0.1532) and ACPA fine-specificity levels (data not shown) did not differ significantly.

### Presence of RA-associated autoantibodies in “seronegative” RA

In a subset of EIRA (*n* = 1944), we have previously investigated the presence of anti-CarP antibodies, which we detected in 43% of RA patients [28]. Now we focused the analysis on the traditionally defined “seronegative” subset of EIRA, i.e. anti-CCP2 IgG−/IgM RF− patients (*n* = 534), and found anti-CarP antibodies in 15.9%, ACPA fine-specificities in 29.8%, and IgA and/or IgG RF in 9.4%, with a co-occurrence of at least two types of RA-associated autoantibodies in 9.6% (Table 3). When combining ACPA, RF, and anti-CarP antibodies, 43.6% of the “seronegative” RA patients were in fact “seropositive”.

**Table 3:** RA-associated autoantibodies in “seronegative” RA

Antibody status^a^	***n***	%
ACPA+/RF+/anti-CarP+	10	1.9
ACPA+/RF+/anti-CarP−	12	2.3
ACPA+/RF−/anti-CarP+	22	4.1
ACPA+/RF−/anti-CarP−	115	21.5
ACPA−/RF+/anti-CarP+	7	1.3
ACPA−/RF+/anti-CarP−	21	3.9
ACPA−/RF−/anti-CarP+	46	8.6
ACPA−/RF−/anti-CarP−	301	56.4

^a^Antibody status was evaluated in 534 anti-CCP2 IgG−/IgM RF− EIRA patients. *ACPA* = any ACPA fine-specificity, *RF* = IgA and/or IgG RF, *anti-CarP* = anti-carbamylated fibrinogen antibodies

### RA-associated autoantibodies in relation to disease course in “seronegative” RA

We then investigated the impact of RA-associated autoantibodies on disease course in “seronegative” RA during a 5-year follow-up period. Compared to patients that were negative for all investigated RA-associated autoantibodies, the presence of ACPA fine-specificities and/or IgG/IgA RF and/or anti-CarP antibodies (in the anti-CCP2−/IgM RF− subset) associated with higher DAS28 during follow-up (Table 4). This observation seemed to be dependent on the presence of ACPA and RF, but not anti-CarP antibodies. Significantly higher DAS28 scores were recorded in the ACPA+/anti-CCP2−/IgM RF− subset (median DAS28: 3.66 versus 1.96, *p* = 0.002) and in the IgA/IgG RF+/anti-CCP2−/IgM RF− subset (median DAS28: 3.17 versus 1.96, *p* = 0.03) at 48 months. Highest DAS28 was found in the ACPA+/anti-CarP−/anti-CCP2−/IgM RF− subset (median DAS28: 3.23 versus 2.14, *p* = 0.03 at 36 months, and 3.69 versus 1.96, *p* = 0.007, at 48 months). Notably, DAS28 was as high (or even higher) in this subset as in the traditionally defined seropositive subset (i.e. anti-CCP2+ and/or IgM RF+). Lowest DAS28 scores during follow-up were noted in ACPA−/anti-CarP+/anti-CCP2−/IgM RF− patients.

**Table 4:** Disease activity during 5-years follow-up, in relation to autoantibody status

RA subset^a^		0 m	3 m	6 m	12 m	24 m	36 m	48 m	60 m
Seronegative	DAS28	5.10	3.49	3.25	2.72	2.33	2.14	1.96	2.2.11
	*n*	163	138	139	154	148	73	47	103
Ab+	DAS28	5.37	3.59	3.34	2.80	2.68	2.63	**2.97**	2.02
	*n*	123	110	107	119	104	56	35	62
ACPA+	DAS28	5.34	3.63	3.41	2.94	2.71	2.99	**3.66**	2.20
	*n*	87	77	79	84	75	40	21	41
RF+	DAS28	5.50	3.67	3.49	2.49	2.27	2.61	**3.17**	1.88
	*n*	27	23	23	26	24	13	8	12
Carb+	DAS18	5.50	3.30	3.35	2.77	2.66	2.13	2.20	1.78
	*n*	43	38	33	40	33	16	14	24
ACPA+/Carb−	DAS28	5.15	3.66	3.44	3.01	2.76	**3.23**	**3.69**	2.37
	*n*	68	61	64	67	60	33	16	32
ACPA−/Carb+	DAS28	5.57	3.14	3.30	2.75	2.74	1.89	1.91	1.71
	*n*	24	22	18	23	18	9	9	15
Seropositive	DAS28	5.08	3.67	3.25	**3.02**	2.71	**2.94**	**2.87**	**2.75**
	*n*	747	650	595	719	685	444	315	522

^a^All RA subsets (with the exception of seropositive RA) are anti-CCP2 IgG−/IgM RF−; Ab+ = ACPA+, and/or IgA RF+, and/or IgG RF+, and/or anti-CarP+; seropositive = anti-CCP2 IgG+ and/or IgM RF+. Median DAS28-CRP values are shown for each RA subset (significantly higher DAS28-CRP compared to seronegative RA in bold). *N* = number of patients in each subset at each time point. 0 m = baseline; 3–60 m = 3 months to 60 months follow-up period

## Discussion

We find autoantibodies in “seronegative” RA, at significantly higher frequencies, levels, and co-occurrence compared to controls, and with a similar pattern of reactivity as in traditionally defined seropositive RA, albeit with a narrower serology in regards to RA-associated autoantibodies. Our data are thus in line with previous studies demonstrating the presence of ACPA in anti-CCP2-negative RA [16, 17, 19] and a recent report, detecting IgA RF and anti-CCP2 IgA in 5.2% of “seronegative” RA [30]. A novel finding in our study is that the extended ACPA/RF serology defines a group of RA patients with a more severe prognosis, and since additional screening of ACPA fine-specificities and IgA/IgG RF was able to identify 35% of the patients in the conventionally defined seronegative RA subset, our data suggest that the use of such extended serology may be clinically useful.

While the presence of ACPA fine-specificities and RF in “seronegative” RA associated with higher disease activity during follow-up, the presence of anti-CarP antibodies associated with lower disease activity in our study. This observation is somewhat contradictory to what has been reported previously, where presence of anti-CarP antibodies was shown to associate with higher disease activity and worse clinical outcome [21, 31, 32]. However, the other studies have either only analysed baseline DAS28, and at baseline, we also found a non-significant trend with higher disease activity in the anti-CarP-positive group, or the other studies have analysed radiological progression over time, while we have analysed DAS28 over time, with no access to radiological data. Moreover, the other studies did not investigate presence of ACPA fine-specificities. Based on our data, nearly 40% of anti-CarP-positive patients within the “seronegative” RA population would also be ACPA-positive. Therefore, it cannot be ruled out that the observed joint destruction in the other studies is primarily associated with ACPA, rather than anti-CarP antibodies.

In accordance with data published by Wagner et al. [17], as well as our recent report [19], we found an association of SE with the presence of ACPA in anti-CCP2-negative RA, suggesting that these patients belong to the same disease entity as anti-CCP2-positive patients. A more detailed analysis of the ACPA fine-specificity response showed that SE associates primarily with some ACPA fine-specificities, not all. These ACPA fine-specificities co-occurred to a large extent, which could possibly indicate cross-reactivity rather than co-occurrence, something that has been shown on a monoclonal level for different ACPA [33–35]. Other ACPA did not co-occur to the same extent, which may suggest different triggering mechanisms for different ACPA fine-specificities. This, we have recently explored in a separate study, demonstrating unique genetic characteristics for different ACPA fine-specificities [36]. We did not find an association between HLA-DRB1 SE and RF in anti-CCP2-negative RA, supporting data generated from single-cell RNA sequencing of RF-positive and ACPA-positive B cells, which suggest a T cell-dependent affinity maturation for the generation of ACPA, but innate immune pathways for the generation of RF [37].

We could not detect an association between smoking and presence of ACPA in anti-CCP2-negative RA, but a significant association with RF, in particular IgA RF. We also observed significantly elevated RF (but not anti-CCP2 IgG) levels in current smokers compared to former smokers. These data are thus in line with recent reports, demonstrating a lack of association between ACPA and smoking in RF-negative RA [38] and that the association between smoking and RA-associated antibodies is likely dependent on RF/RF levels [39]. The association between smoking and IgA RF is well known [40, 41] and points to the putative role of mucosal inflammation in the generation of IgA RF. Notably, RF has been reported in healthy smokers [41, 42].

Contrary to ACPA and RF, the other autoantibodies—not primarily associated with RA—were low in frequency, equally distributed among anti-CCP2-positive and anti-CCP2-negative RA patients, and showed no associations with genetic and environmental risk factors. Most common were anti-R060/SSA and anti-Ro52/SSA antibodies, each with a frequency of around 5%, similar to previous reports [43]. Their presence may indicate clinical features associated with other systemic inflammatory diseases, in addition to classical RA symptoms.

Although the PTPN22 polymorphism (rs2476601) was more frequent among anti-CCP2-positive patients compared to anti-CCP2-negative, we found a significant association with both subsets, similar to previous reports [44, 45]. Presence/absence of ACPA fine-specificities, RF, and other autoantibodies did not affect this positive association. PTPN22 polymorphism has been described as a risk factor for several autoimmune diseases [46], and healthy carriers have been reported to have more autoreactive B cells compared to non-carriers [47]. Hence, the association of PTPN22 with autoantibody-negative RA was a bit surprising and potentially suggests the presence of yet other autoimmune reactions.

Our study is by no means complete when it comes to the analysis of RA-associated autoantibodies. We did not investigate the presence of anti-CCP2 IgA [48] nor did we analyse presence of ACPA using other commercial clinical tests, like the CCP3/CCP3.1 assays or the mutated citrullinated vimentin (MCV) assay. The antibody response against human PAD enzymes [49] and acetylated proteins [50] was not investigated either, and anti-CarP antibodies were analysed using carbamylated fibrinogen as an antigen [28], while other studies have used carbamylated foetal calf serum [21, 39].

Another point to be considered is the loss of specificity when analysing multiple autoantibody reactivities. Hence, for a diagnostic setting, highly specific cutoff values should be strictly applied to each individual antibody and be based not only on population controls but on disease controls with other rheumatic and autoimmune conditions. Still, our data suggest that the number of ACPA fine-specificities, as well as co-occurrence of ACPA and IgA/IgG RF, could be useful diagnostic markers.

## Conclusion

Our study confirms that seronegative RA, defined only from the presence of anti-CCP2 IgG and IgM RF, is not truly a seronegative disease subset. Presence of ACPA and RF in the conventionally defined seronegative RA population defines a group of patients that resemble seropositive patients with respect to risk factors and clinical picture. Thus, our data suggest that extended ACPA and RF serology could be clinically useful in order to capture patients early in the disease process and to identify a subset with a high need for active treatment.

## Supplementary information

**Additional file 1: Supplementary Table 1.** Citrullinated peptide antigens on the multiplex microarray. The table lists citrullinated antigens used on the multiplex microarray, including name, protein of origin, and amino acid sequence, as well as references.

**Additional file 2: Supplementary Table 2.** Other (non-citrullinated) autoantigens on the multiplex microarray. The table lists the other antigens used on the multiplex microarray, including name, protein of origin, and major associated disease(s).

**Additional file 3: Supplementary Table 3.** Baseline characteristics in CCP2-positive and -negative RA. The table show baseline characteristics for the EIRA patients included in the study, based on anti-CCP2 IgG status; *p*-values indicate differences between subsets with respect to: age, female-to-male ratio, number of smokers, HLA-DRB1 SE-positivity, PTPN22-positivity, DAS28 and CRP.

**Additional file 4: Supplementary Figure 1.** ACPA fine-specificity levels in anti-CCP2-negative RA and controls. ACPA levels for 19 individual ACPA fine-specificities are shown in separate graphs with AU values on the Y-axes. Only ACPA levels above cutoff for positivity are shown.

**Additional file 5: Supplementary Table 4.** Other autoantibodies in anti-CCP2-positive and -negative RA and controls. Frequencies of 17 other autoantibodies in anti-CCP2-positive RA, anti-CCP2-negative RA and controls are shown, as well as median antibody levels in anti-CCP2-positive and -negative RA (*p*-values indicate differences between anti-CCP2-negative and anti-CCP2-positive RA subsets).

**Additional file 6: Supplementary Table 5.** Associations between different autoantibodies and PTPN22 polymorphism, in anti-CCP2-negative RA. Odds ratios with 95% confidence intervals are shown for associations between PTPN22 polymorphism and presence/absence of ACPA, RF, other autoantibodies, or "any autoantibody" in anti-CCP2-negative RA.

**Additional file 7: Supplementary Table 6.** Associations between different ACPA fine-specificities and HLA-DRB1 SE, in anti-CCP2-positive and anti-CCP2-negative RA. Odds ratios with 95% confidence intervals are shown for associations between HLA-DRB1 SE and presence/absence of 19 different ACPA fine-specificities, in anti-CCP2-positive and -negative RA: p-values indicate differences in ORs between ACPA fine-specificity-positive and -negative subsets in the anti-CCP2-positive subset.

**Additional file 8: Supplementary Table 7.** Associations between RF isotypes and smoking, in anti-CCP2-positive and anti-CCP2-negative RA. Odds ratios with 95% confidence intervals are shown for associations between smoking and presence/absence of IgM, IgG or IgA RF, in anti-CCP2-positive and -negative RA; p-values indicate differences in ORs between RF isotype-positive and -negative subsets.

**Additional file 9: Supplementary Figure 2.** Antibody levels in EIRA RA cases based on smoking status. IgM, IgG and IgA RF levels, as well as anti-CCP2 IgG levels, are shown for never, current and former smokers in all EIRA RA patients where smoking data was available.

## Data Availability

All data generated during this study are included in this published article, and in its supplementary information files, or are available from the corresponding author on reasonable request. The EIRA dataset on which the current study is based is deposited in the EIRA database at Karolinska Institutet, Stockholm, Sweden.

## Abbreviations

ACPA: Anti-citrullinated protein antibody
RA: Rheumatoid arthritis
RF: Rheumatoid factor
ACR: American College of Rheumatology
EULAR: European League Against Rheumatism
SE: Shared epitope
CCP: Cyclic citrullinated peptide
CII: Type II collagen
Anti-CarP antibody: Anti-carbamylated protein antibody
EIRA: Epidemiological investigation of RA
DAS28: Disease activity score for 28 joints
CRP: C-reactive protein
hnRNP-A3: Heterogeneous nuclear ribonucleoprotein-A3
OR: Odds ratio
CI: Confidence interval
MCV: Mutated citrullinated vimentin
