# Supplementary material for: Presence of autoantibodies in “seronegative” rheumatoid arthritis associates with classical risk factors and high disease activity
Source: Arthritis Res Ther. 2020 Jul 16;22:170. doi: 10.1186/s13075-020-02191-2 (PMC7364538; doi:10.1186/s13075-020-02191-2)
Supplement: Supplementary file 1 — Additional file 1: Supplementary Table 1. Citrullinated peptide antigens on the multiplex microarray. The table lists citrullinated antigens used on the multiplex microarray, including name, protein of origin, and amino acid sequence, as well as references. [file 13075_2020_2191_MOESM1_ESM.pdf]

Supplementary table 1 Citrullinated peptide antigens on the multiplex microarray

Antigen	Protein	(amino acids)	Amino acid sequence	References
Cit-peptide 1	hnRNP-A3	pending patent application		*
Cit-peptide 5	hnRNP-A3	pending patent application		*
Cit-peptide Bla26	hnRNP-A3	pending patent application		*
Cit-peptide Z1	hnRNP-A3	pending patent application		*
Cit-peptide Z2	hnRNP-A3	pending patent application		*
Cit-C1	CII	(355-378)	$(GPO)_5$-GLOGACITGLTGCITOGDAGPQGKVGPS-$(GPO)_2$-GKKYG	1
Cit-F4$_{(Cit-Cit)}$	CII	(916-939)	$(GPO)_5$-GDKGEAGEOGECITGLKGHCITGFTGLQ-$(GPO)_2$-GKKYG	2
Cit-F4$_{(Cit-R)}$	CII	(916-939)	$(GPO)_5$-GDKGEAGEOGECITGLKGHRGFTGLQ-$(GPO)_2$-GKKYG	2
Cit-F4$_{(R-Cit)}$	CII	(916-939)	$(GPO)_5$-GDKGEAGEOGERGLKGHCITGFTGLQ-$(GPO)_2$-GKKYG	2
CEP-1	α-enolase	(5-21)	C-KIHACITEIFDSCITGNPTVE-C	3
Cit-Fibα$_{36-50}$	fibrinogen α	(36-50)	GPCITVVECITHQSACKDS	4-6
Cit-Fibα$_{563-583}$	fibrinogen α	(563-583)	HHPGIAEFPSCITGKSSSYSKQF	7, 8
Cit-Fibα$_{580-600}$	fibrinogen α	(580-600)	SKQFTSSTSYNCITGDSTFESKS	8, 9
Cit-Fib-α$_{621-635}$	fibrinogen α	(621-635)	CITGHAKSCITPVCITGIHTS	4-6
Cit-Fibß$_{36-52}$	fibrinogen ß	(36-52)	NEEGFFSACITGHRPLDKK	10
Cit-Fibß$_{60-74}$	fibrinogen ß	(60-74)	CITPAPPPISGGGYCITACIT	4-6
cfc1-cyc (CCP1)	filaggrin	(304-324)	HQCHQESTCITGRSRGRCGRSGS	11
Cit-Vim$_{2-17}$	vimentin	(2-17)	STCITSVSSSSYCITCITMFGG	12
Cit-Vim$_{60-75}$	vimentin	(60-75)	VYATCITSSAVCITLCITSSVP	10

* Karl Skriner, Department of Rheumatology and Clinical Immunology, Charité University, Berlin, Germany

CII = collagen type II; Cit/CIT = citrullinated/citrulline; hnRNP-A3 = heterogeneous nuclear ribonucleoprotein A3

REFERENCES (supplementary table 1)

1. Burkhardt H, Sehnert B, Bockermann R, Engström A, Kalden JR, Holmdahl R. Humoral immune response to citrullinated collagen type II determinants in early rheumatoid arthritis. Eur J Immunol 2005, May;35(5):1643-52.

2. Haag S, Schneider N, Mason DE, Tuncel J, Andersson IE, Peters EC et al. Identification of new citrulline-specific autoantibodies, which bind to human arthritic cartilage, by mass spectrometric analysis of citrullinated type II collagen. Arthritis Rheumatol 2014, Jun;66(6):1440-9.

3. Lundberg K, Kinloch A, Fisher BA, Wegner N, Wait R, Charles P et al. Antibodies to citrullinated alpha-enolase peptide 1 are specific for rheumatoid arthritis and cross-react with bacterial enolase. Arthritis Rheum 2008, 58:3009-3019.

4. Sebbag M, Moinard N, Auger I, Clavel C, Arnaud J, Nogueira L et al. Epitopes of human fibrin recognized by the rheumatoid arthritis-specific autoantibodies to citrullinated proteins. Eur J Immunol 2006, 36:2250-2263.

5. Iobagiu C, Magyar A, Nogueira L, Carnillet M, Sebbag M, Arnaud J et al. The antigen specificity of the rheumatoid arthritis- associated ACPA directed to citrullinated fibrin is very closely restricted. J Autoimmun 2011, 37:263-272.

6. Cornillet M, Sebbag M, Verrouil E, Magyar A, Babos F, Ruyssen-Witrand A et al. The fibrin-derived citrullinated peptide β60-74Cit$_{60,72,74}$ bears the major ACPA epitope recognised by the Rheumatoid Arthritis-specific anti-citrullinated fibrinogen autoantibodies and anti-CCP2 antibodies. Ann Rheum Dis 73:1246-52, 2014.

7. Hermansson M, Artemenko K, Ossipova E, Eriksson H, Lengqvist J, Makrygiannakis D et al. MS analysis of rheumatoid arthritic synovial tissue identifies specific citrullination sites on fibrinogen. Proteom Clin Appl 2010, 4:511-518

8. Too CL, Murad S, Hansson M, Alm LM, Dhaliwal JS, Holmdahl R et al. Spectrum of Anti Citrullinated Peptide Antibodies (ACPA) fine specificities is different between Malaysian and Swedish rheumatoid arthritis patients: Implications for pathogenesis of RA. Arthritis Rheumatol 2016, Epub 2016/08/03

9. Hansson M, Mathsson L, Schlederer T, Israelsson L, Matsson P, Nogueira L et al. Validation of a multiplex chip-based assay for the detection of autoantibodies against citrullinated peptides. Arthritis Res Ther 2012, Oct 1;14(5):R201.

10. Verpoort KN, Cheung K, Ioan-Facsinay A, van der Helm-van Mil AH, de Vries-Bouwstra JK, Allaart CF et al. Fine specificity of the anti-citrullinated protein antibody response is influenced by the shared epitope alleles. Arthritis Rheum 2007, 56:3949-3952.

11. Schellekens GA, Visser H, de Jong BA, van den Hoogen FH, Hazes JM, Breeveld FC et al. The diagnostic properties of rheumatoid arthritis antibodies recognizing a cyclic citrullinated peptide. Arthritis Rheum 2000, 43:155-163.

12. Snir O, Widhe M, Hermansson M, von Spee C, Lindberg J, Hensen S et al. Antibodies to several citrullinated antigens are enriched in the joints of rheumatoid arthritis patients. Arthritis Rheum 2010, 62:44-52.
