# Supplementary material for: Presence of autoantibodies in “seronegative” rheumatoid arthritis associates with classical risk factors and high disease activity
Source: Arthritis Res Ther. 2020 Jul 16;22:170. doi: 10.1186/s13075-020-02191-2 (PMC7364538; doi:10.1186/s13075-020-02191-2)
Supplement: Supplementary file 2 — Additional file 2: Supplementary Table 2. Other (non-citrullinated) autoantigens on the multiplex microarray. The table lists the other antigens used on the multiplex microarray, including name, protein of origin, and major associated disease(s). [file 13075_2020_2191_MOESM2_ESM.pdf]

Supplementary table 2 Other (non-citrullinated) autoantigens on the multiplex microarray

Antigen	Protein of origin	Major associated disease(s)
Ro60/SSA	RNA associated protein SS-A/Ro (60kD)	SLE, Sjögren's syndrome
Ro52/SSA	RNA associated protein SS-A/Ro (52kD)	SLE, Sjögren's syndrome
La/SSB	RNA associated protein SS-B/La	SLE, Sjögren's syndrome
U1 RNP-70	U1 RNP complex (small nuclear ribonucleoprotein, 70kD)	MCTD, SLE
U1 RNP-A	U1 RNP complex (small nuclear ribonucleoprotein A)	MCTD, SLE
U1 RNP-C	U1 RNP complex (small nuclear ribonucleoprotein C)	MCTD, SLE
SmD	U1 RNP complex (SmD peptide)	SLE
SmBB	U1 RNP complex (SmBB protein)	SLE
dsDNA	Double stranded DNA	SLE
PCNA	Proliferating cell nuclear antigen	SLE
Rip P2	Ribosomal protein P2	SLE
Scl70	Topoisomerase-1 (Scl protein, 70kD)	Systemic sclerosis
CENP B	Centromere protein B	Limited scleroderma (CREST syndrome)
PMScl100	Exosome (PM-Scl protein, 100kD)	Polymyosits/ scleroderma overlap syndrome
RNA pol III	RNA polymerase III	Systemic sclerosis
Fibrillarin	Fibrillarin	Systemic sclerosis
Jo 1	Histidyl-tRNA synthetase	Polymyositis/dermatomyositis, anti-synthetase syndrome

CREST = calcinosis, Raynaud phenomenon, esophageal dysmotility, sclerodactyly, and telangiectasia; MCTD = mixed connective tissue disease; PM-Scl = polymyosit-scleroderma antigen; SLE = systemic lupus erythematosus; Scl = scleroderma antigen; Sm = Smith antigen; SSA = Sjögren's syndrome type A antigen; SSB = Sjögren's syndrome type B antigen
