# Supplementary material for: Presence of autoantibodies in “seronegative” rheumatoid arthritis associates with classical risk factors and high disease activity
Source: Arthritis Res Ther. 2020 Jul 16;22:170. doi: 10.1186/s13075-020-02191-2 (PMC7364538; doi:10.1186/s13075-020-02191-2)
Supplement: Supplementary file 3 — Additional file 3: Supplementary Table 3. Baseline characteristics in CCP2-positive and -negative RA. The table show baseline characteristics for the EIRA patients included in the study, based on anti-CCP2 IgG status; p-values indicate differences between subsets with respect to: age, female-to-male ratio, number of smokers, HLA-DRB1 SE-positivity, PTPN22-positivity, DAS28 and CRP. [file 13075_2020_2191_MOESM3_ESM.pdf]

Supplementary table 3 Baseline characteristics in anti-CCP2-positive and -negative RA

Characteristics	CCP2+	CCP2-	P-value
**Age, median years (range)** (N: 1,766 CCP2+ / 989 CCP2-)	54 (18-70)	57 (18-70)	**<0.0001**
**Female, n (%)** (N: 1,762 CCP2+ / 984 CCP2-)	1,261 (72)	701 (71)	0.9
**Ever Smoker[a], n (%)** (N: 1,385 CCP2+ / 813 CCP2-)	1007 (72.7)	508 (62.5)	**<0.0001**
***HLA-DRB1* SE, n (%)** (N: 1,385 CCP2+ / 813 CCP2-)	1,180 (85.2)	441 (54.2)	**<0.0001**
***PTPN22,* n (%)** (N: 1,385 CCP2+ / 813 CCP2-)	427 (30.8)	222 (27.3)	**0.02**
**DAS28, median (range)** (N: 1,282 CCP2+ / 704 CCP2-)	5.11 (0.03-8.89)	5.21 (0.03-8.37)	0.053
**CRP, median mg/L (range)** (N: 1,357 CCP2+ / 739 CCP2-)	12 (0-332)	11 (0-221)	0.208

[a] Ever smoker includes current and former smokers. Significant P-values are shown in bold. CCP = cyclic citrullinated peptide; CRP = C-reactive protein; DAS28 = disease activity score using 28 joint counts; HLA = human leukocyte antigen; PTPN22 = protein tyrosine phosphatase non-receptor type 22; SE = shared epitope.
