# Supplementary material for: Presence of autoantibodies in “seronegative” rheumatoid arthritis associates with classical risk factors and high disease activity
Source: Arthritis Res Ther. 2020 Jul 16;22:170. doi: 10.1186/s13075-020-02191-2 (PMC7364538; doi:10.1186/s13075-020-02191-2)
Supplement: Supplementary file 4 — Additional file 4: Supplementary Figure 1. ACPA fine-specificity levels in anti-CCP2-negative RA and controls. ACPA levels for 19 individual ACPA fine-specificities are shown in separate graphs with AU values on the Y-axes. Only ACPA levels above cutoff for positivity are shown. [file 13075_2020_2191_MOESM4_ESM.pdf]

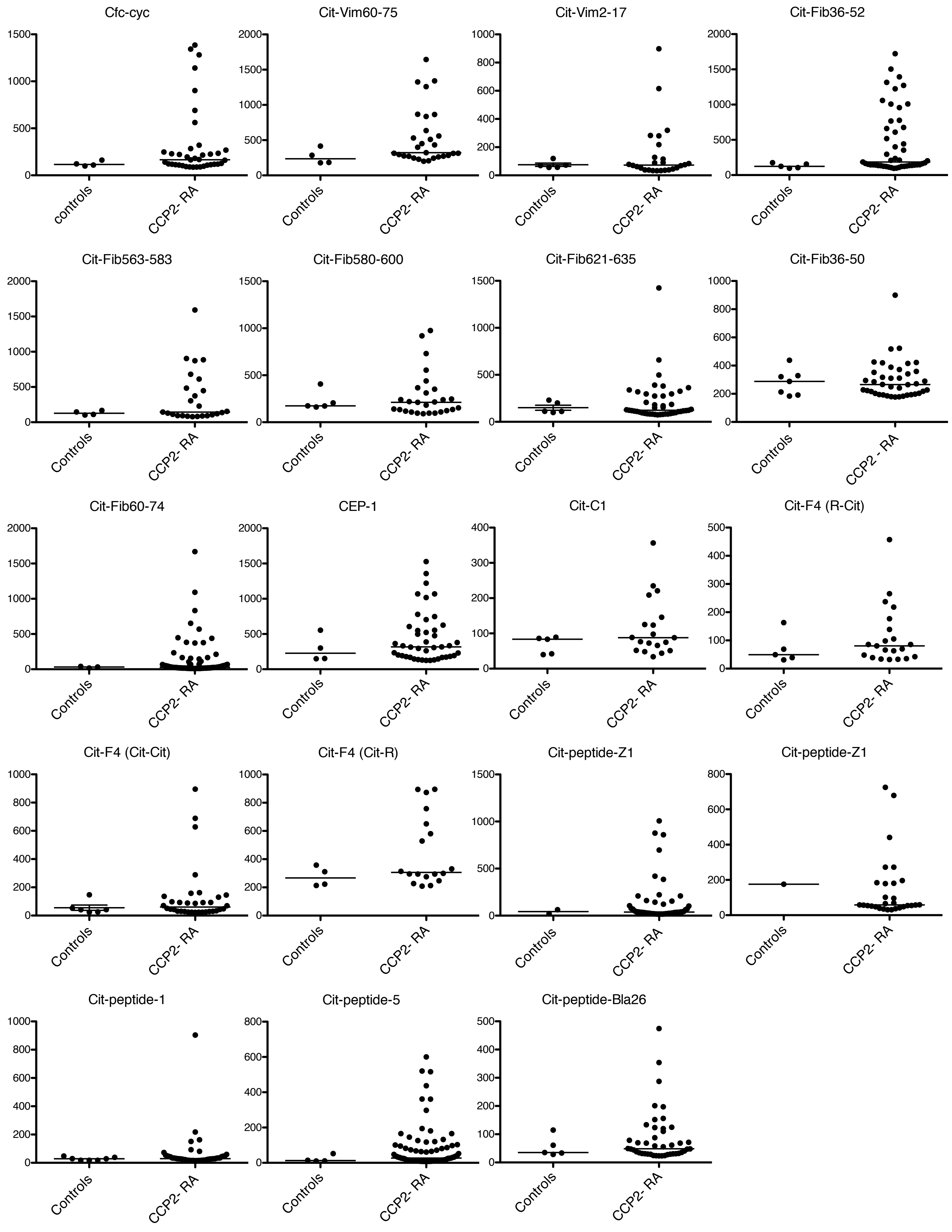
Cfc-cyc
0
500
1000
1500
controls
CCP2- RA
Cit-Vim60-75
0
500
1000
1500
2000
Controls
CCP2- RA
Cit-Vim2-17
0
200
400
600
800
1000
Controls
CCP2- RA
Cit-Fib36-52
0
500
1000
1500
2000
Controls
CCP2- RA
Cit-Fib563-583
0
500
1000
1500
2000
Controls
CCP2- RA
Cit-Fib580-600
0
500
1000
1500
Controls
CCP2- RA
Cit-Fib621-635
0
500
1000
1500
Controls
CCP2- RA
Cit-Fib36-50
0
200
400
600
800
1000
Controls
CCP2 - RA
Cit-Fib60-74
0
500
1000
1500
2000
Controls
CCP2- RA
CEP-1
0
500
1000
1500
2000
Controls
CCP2- RA
Cit-C1
0
100
200
300
400
Controls
CCP2- RA
Cit-F4 (R-Cit)
0
100
200
300
400
500
Controls
CCP2- RA
Cit-F4 (Cit-Cit)
0
200
400
600
800
1000
Controls
CCP2- RA
Cit-F4 (Cit-R)
0
200
400
600
800
1000
Controls
CCP2- RA
Cit-peptide-Z1
0
500
1000
1500
Controls
CCP2- RA
Cit-peptide-Z1
0
200
400
600
800
Controls
CCP2- RA
Cit-peptide-1
0
200
400
600
800
1000
Controls
CCP2- RA
Cit-peptide-5
0
200
400
600
800
Controls
CCP2- RA
Cit-peptide-Bla26
0
100
200
300
400
500
Controls
CCP2- RA

Supplementary figure 1 ACPA fine-specificity levels in anti-CCP2-negative RA and controls. ACPA levels for nineteen individual ACPA fine-specificities are shown in separate graphs with AU (arbitrary units) values on the Y-axes. Only ACPA levels above cutoff for positivity are shown. Horizontal lines indicate median for the group (n = 1 - 7 controls and n = 19 - 92 anti-CCP2-negative RA patients). Differences in ACPA levels between ACPA-positive anti-CCP2-negative RA patients and controls did not reach statistical significance.
