# Supplementary material for: Presence of autoantibodies in “seronegative” rheumatoid arthritis associates with classical risk factors and high disease activity
Source: Arthritis Res Ther. 2020 Jul 16;22:170. doi: 10.1186/s13075-020-02191-2 (PMC7364538; doi:10.1186/s13075-020-02191-2)
Supplement: Supplementary file 5 — Additional file 5: Supplementary Table 4. Other autoantibodies in anti-CCP2-positive and -negative RA and controls. Frequencies of 17 other autoantibodies in anti-CCP2-positive RA, anti-CCP2-negative RA and controls are shown, as well as median antibody levels in anti-CCP2-positive and -negative RA (p-values indicate differences between anti-CCP2-negative and anti-CCP2-positive RA subsets). [file 13075_2020_2191_MOESM5_ESM.pdf]

Supplementary Table 4 Other autoantibodies in anti-CCP2-positive and -negative RA and controls

**Antigen**	**Antibody frequencies** (%)[a]				**Antibody levels** (median)		
	CCP2+	CCP2-	controls	P-value[b]	CCP2+	CCP2-	P-value[c]
Ro60/SSA	**4.8**	**5.3**	1.6	0.61	419.0	712.8	**0.003**
Ro52/SSA	**3.4**	**5.3**	1.4	**0.02**	1252.8	1320.4	**0.073**
PMScl100	3.1	**4.9**	2.2	**0.02**	156.8	154.1	0.471
dsDNA	2.8	3.9	1.9	0.99	25.5	25.4	0.886
La/SSB	**3.1**	2.7	1.1	0.63	232.6	381.2	0.045
U1 RNP-C	2.1	**3.6**	1.6	**0.02**	107.2	147.2	**0.008**
Fibrillarin	3.1	1.8	1.6	**0.04**	29.0	33.2	0.838
CENPB	**2.1**	2.0	0.5	0.90	116.2	123.5	0.628
Jo1	2.3	1.5	1.4	0.18	92.1	126.2	0.597
Rip P2	1.6	2.5	1.9	0.08	111.8	76.8	**0.05**
U1 RNP-A	1.2	2.7	1.4	**0.003**	377.1	631.8	**0.002**
U1 RNP-70	2.0	0.7	2.4	**0.005**	67.9	71.2	0.578
RNA pol III	1.5	1.6	1.6	0.77	436.1	427.1	0.776
Scl70	1.5	1.3	0.8	0.74	197.7	194.4	0.439
SmD	1.4	1.0	1.9	0.43	404.0	413.0	0.762
PCNA	0.8	1.7	1.6	0.41	196.5	241.6	0.317
SmBB	0.5	1.0	1.6	0.13	223.6	185.2	0.221

[a] Bold figures indicate significantly higher antibody frequencies than in controls. P-values show differences in [b] antibody frequencies and [c] antibody levels, between anti-CCP2-positive and anti-CCP2-negative RA.
