# Supplementary material for: Presence of autoantibodies in “seronegative” rheumatoid arthritis associates with classical risk factors and high disease activity
Source: Arthritis Res Ther. 2020 Jul 16;22:170. doi: 10.1186/s13075-020-02191-2 (PMC7364538; doi:10.1186/s13075-020-02191-2)
Supplement: Supplementary file 6 — Additional file 6: Supplementary Table 5. Associations between different autoantibodies and PTPN22 polymorphism, in anti-CCP2-negative RA. Odds ratios with 95% confidence intervals are shown for associations between PTPN22 polymorphism and presence/absence of ACPA, RF, other autoantibodies, or "any autoantibody" in anti-CCP2-negative RA. [file 13075_2020_2191_MOESM6_ESM.pdf]

Supplementary Table 5 Associations between different autoantibodies and PTPN22 polymorphism, in anti-CCP2-negative RA

	Exposure			
**Subgroup**	***PTPN22-***	***PTPN22+***	**OR (95% CI)**[a]	**OR (95% CI)**[b]
Controls	2202	595	1.0 (ref)	1.0 (ref)
ACPA-	370	141	**1.43** (1.15-1.77)	**1.42** (1.15-1.76)
ACPA+	221	81	**1.36** (1.04-1.79)	**1.38** (1.05-1.80)
RF-	406	145	**1.33** (1.08-1.65)	**1.34** (1.08-1.65)
RF+	185	77	**1.55** (1.17-2.06)	**1.55** (1.17-2.06)
other Ab-	404	154	**1.42** (1.16-1.75)	**1.43** (1.16-1.76)
other Ab+	187	68	**1.35** (1.01-1.81)	**1.34** (1.00-1.80)
any Ab-	176	71	**1.51** (1.13-2.02)	**1.51** (1.13-2.02)
any Ab+	410	148	**1.36** (1.10-1.67)	**1.36** (1.10-1.68)

Odds ratios were adjusted for [a] age, gender, residential area, and [b] SE and smoking. ACPA = any ACPA fine-specificity; RF = IgM and/or IgG and/or IgA RF; other Ab = any other autoantibody; any Ab = ACPA and/or RF and/or other autoantibody.
