# Supplementary material for: Presence of autoantibodies in “seronegative” rheumatoid arthritis associates with classical risk factors and high disease activity
Source: Arthritis Res Ther. 2020 Jul 16;22:170. doi: 10.1186/s13075-020-02191-2 (PMC7364538; doi:10.1186/s13075-020-02191-2)
Supplement: Supplementary file 7 — Additional file 7: Supplementary Table 6. Associations between different ACPA fine-specificities and HLA-DRB1 SE, in anti-CCP2-positive and anti-CCP2-negative RA. Odds ratios with 95% confidence intervals are shown for associations between HLA-DRB1 SE and presence/absence of 19 different ACPA fine-specificities, in anti-CCP2-positive and -negative RA: p-values indicate differences in ORs between ACPA fine-specificity-positive and -negative subsets in the anti-CCP2-positive subset. [file 13075_2020_2191_MOESM7_ESM.pdf]

Supplementary table 6 Associations between different ACPA fine-specificities and HLA-DRB1 SE, in anti-CCP2-positive and anti-CCP2-negative RA

		OR (95% CI)[a]		
**Subgroup**		**CCP2-**	**CCP2+**	**P-value**[b]
Cit-Fibß$_{60\text{-}74}$	neg	1.0 (0.8-1.2)	**3.8** (2.5-4.6)	
	pos	**2.2** (1.4-3.7)	**6.0** (5.0-7.2)	**0.0007**
Cit-Peptide-5	neg	1.0 (0.9-1.2)	**4.0** (3.0-5.4)	
	pos	1.5 (0.9-2.4)	**5.8** (4.8-7.0)	**0.04**
Cit-Peptide-Z1	neg	1.0 (0.9-1.2)	**3.3** (2.6-4.2)	
	pos	1.5 (0.8-2.8)	**7.0** (5.5-8.4)	**<0.0001**
Cit-Fibß$_{36\text{-}52}$	neg	1.0 (0.9-1.3)	**5.8** (4.4-7.7)	
	pos	1.0 (0.5-1.9)	**5.1** (4.2-6.2)	0.4
Cit-Vim$_{60\text{-}75}$	neg	1.0 (0.9-1.2)	**3.1** (2.5-3.9)	
	pos	**2.8** (1.1-7.0)	**7.9** (6.3-10.0)	**<0.0001**
CEP-1	neg	1.0 (0.9-1.2)	**3.4** (2.7-4.2)	
	pos	1.8 (0.9-3.7)	**7.7** (6.1-9.7)	**<0.0001**
cfc1-cyc	neg	1.1 (0.9-1.3)	**5.9** (4.6-7.7)	
	pos	0.9 (0.4-2.1)	**5.0** (4.1-6.1)	0.2
Cit-Fibα$_{563\text{-}583}$	neg	1.1 (0.9-1.2)	**4.5** (3.5-5.6)	
	pos	1.3 (0.5-3.3)	**6.0** (4.9-7.5)	0.06
Cit-Peptide-Z2	neg	1.1 (0.9-1.2)	**4.7** (3.8-5.9)	
	pos	1.5 (0.6-3.4)	**5.9** (4.7-7.5)	0.2
Cit-Peptide-1	neg	1.1 (0.9-1.2)	**4.7** (3.9-5.8)	
	pos	1.3 (0.7-2.4)	**6.1** (4.8-7.8)	0.07
Cit-Fibα$_{621\text{-}635}$	neg	1.0 (0.9-1.2)	**4.9** (4.0-6.0)	
	pos	1.5 (0.8-2.9)	**5.9** (4.6-7.5)	0.3
Cit-Peptide-Bla26	neg	1.1 (0.9-1.3)	**4.7** (3.9-5.8)	
	pos	0.6 (0.3-1.2)	**6.3** (4.9-8.2)	0.1
Cit-Vim$_{2\text{-}17}$	neg	1.1 (0.9-1.2)	**4.4** (3.6-5.4)	
	pos	1.3 (0.6-3.1)	**7.1** (5.4-9.2)	**0.007**
Cit-F4$_{(Cit\text{-}Cit)}$	neg	1.0 (0.9-1.3)	**5.7** (4.6-7.0)	
	pos	0.8 (0.4-1.7)	**4.8** (3.7-6.1)	0.2
Cit-F4$_{(R\text{-}Cit)}$	neg	1.1 (0.9-1.2)	**5.2** (4.3-6.3)	
	pos	1.3 (0.5-3.7)	**5.6** (4.2-7.4)	0.8
Cit-Fibα$_{580\text{-}600}$	neg	1.1 (0.9-1.2)	**4.8** (4.0-5.8)	
	pos	1.4 (0.6-3.3)	**7.2** (5.1-10.1)	0.06
Cit-Fibα$_{36\text{-}50}$	neg	1.1 (0.9-1.3)	**5.5** (4.6-6.7)	
	pos	0.9 (0.4-1.9)	**4.4** (3.1-6.1)	0.2
Cit-C1	neg	1.1 (0.9-1.2)	**5.2** (4.4-6.2)	
	pos	2.2 (0.7-7.2)	**5.9** (3.7-9.3)	0.6
Cit-F4$_{(Cit\text{-}R)}$	neg	1.1 (0.9-1.2)	**5.8** (4.9-6.9)	
	pos	2.3 (0.8-6.5)	**2.1** (1.4-3.4)	**<0.0001**

[a] Odds ratios (OR) were adjusted for age, gender and residential area. Significant ORs are shown in bold. [b] P-values indicate differences in ORs between ACPA fine-specificity-positive and -negative subsets, in anti-CCP2-positive RA.
