# Supplementary material for: Presence of autoantibodies in “seronegative” rheumatoid arthritis associates with classical risk factors and high disease activity
Source: Arthritis Res Ther. 2020 Jul 16;22:170. doi: 10.1186/s13075-020-02191-2 (PMC7364538; doi:10.1186/s13075-020-02191-2)
Supplement: Supplementary file 8 — Additional file 8: Supplementary Table 7. Associations between RF isotypes and smoking, in anti-CCP2-positive and anti-CCP2-negative RA. Odds ratios with 95% confidence intervals are shown for associations between smoking and presence/absence of IgM, IgG or IgA RF, in anti-CCP2-positive and -negative RA; p-values indicate differences in ORs between RF isotype-positive and -negative subsets. [file 13075_2020_2191_MOESM8_ESM.pdf]

Supplementary table 7 Associations between RF isotypes and smoking, in anti-CCP2-positive and anti-CCP2-negative RA

**Subgroup**	**Exposure**			
**CCP2+**	**Never smoker**	**Ever smokers**	**OR** (95% CI)[a]	**P-value**[b]
Controls	1280	1589	1.0 (ref)	
RF IgM-	39	86	**1.8** (1.2-2.6)	0.3
RF IgM+	339	920	**2.2** (1.8-2.5)	
RF IgG-	115	200	**1.4** (1.1-1.9)	**<0.0001**
RF IgG+	263	806	**2.4** (2.0-2.8)	
RF IgA-	218	360	**1.4** (1.1-1.7)	**<0.0001**
RF IgA+	160	646	**3.1** (2.5-3.8)	
**CCP2-**				
Controls	1208	1589	1.0 (ref)	
RF IgM-	247	359	1.1 (0.9-1.3)	**0.002**
RF IgM+	57	142	**1.9** (1.4-2.7)	
RF IgG-	258	400	**1.2** (1.0-1.4)	0.07
RF IgG+	46	101	**1.7** (1.2-2.4)	
RF IgA-	281	435	**1.2** (1.0-1.4)	**0.01**
RF IgA+	23	66	**2.2** (1.4-3.6)	

[a] Odds ratios (OR) were adjusted for age, gender, residential area, SE and PTPN22. Significant ORs are shown in bold. [b] P-values indicate differences in ORs between RF isotype-positive and -negative RA subsets.
