# Supplementary material for: Presence of autoantibodies in “seronegative” rheumatoid arthritis associates with classical risk factors and high disease activity
Source: Arthritis Res Ther. 2020 Jul 16;22:170. doi: 10.1186/s13075-020-02191-2 (PMC7364538; doi:10.1186/s13075-020-02191-2)
Supplement: Supplementary file 9 — Additional file 9: Supplementary Figure 2. Antibody levels in EIRA RA cases based on smoking status. IgM, IgG and IgA RF levels, as well as anti-CCP2 IgG levels, are shown for never, current and former smokers in all EIRA RA patients where smoking data was available. [file 13075_2020_2191_MOESM9_ESM.pdf]

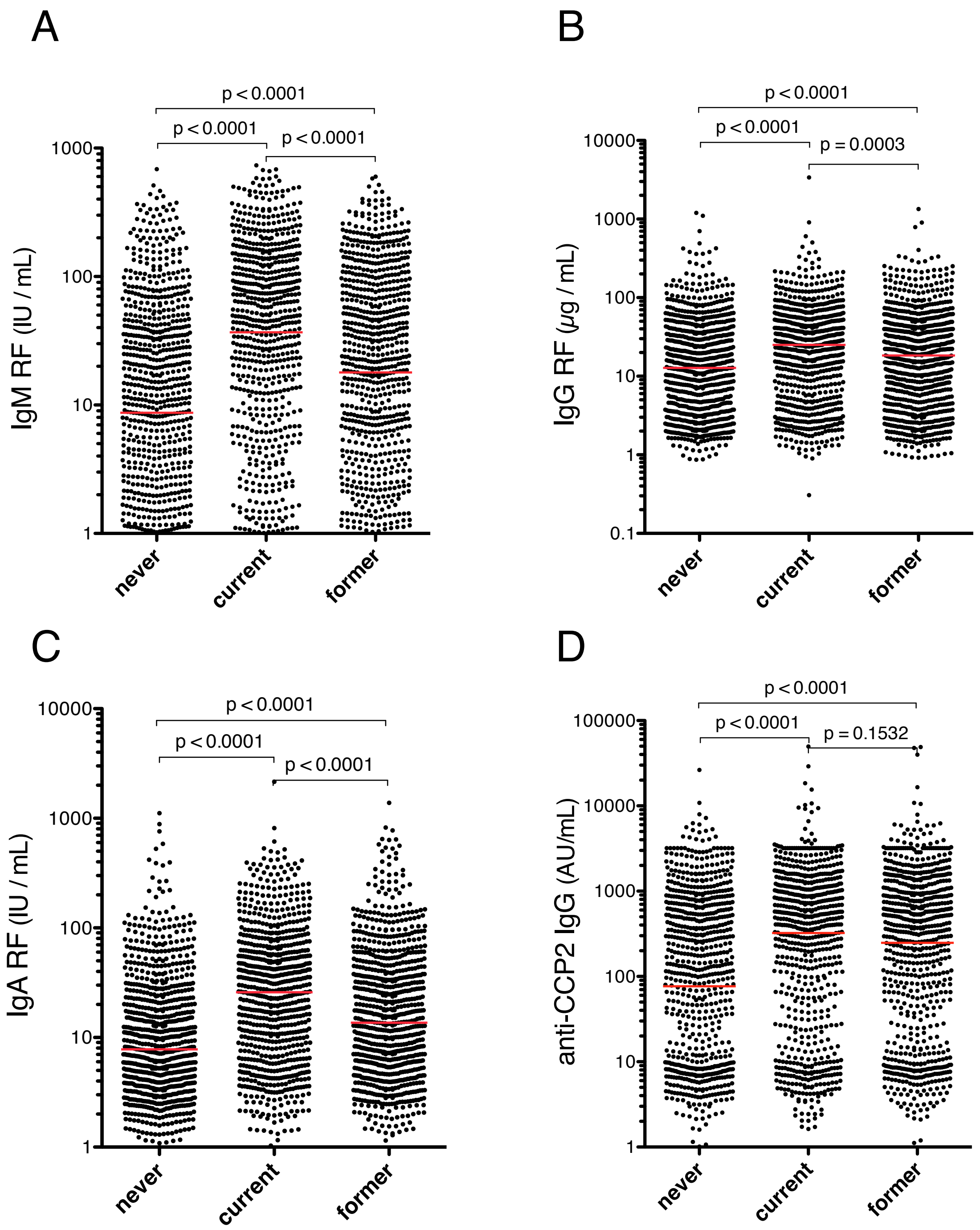

Supplementary figure 2 Antibody levels in EIRA RA cases based on smoking status. IgM RF (A), IgG RF (B) and IgA RF (C) levels were significantly increased in current and former smokers compared to never smokers, and in current smokers compared to former smokers. Anti-CCP2 IgG levels (D) were significantly increased in current and former smokers compared to never smokers, but not in current smokers compared to former smokers. The red horizontal lines indicate median antibody levels. Y-axes show antibody levels on a logarithmic scale. Never = never smoked; current = current smoker; former = former smoker.
